# Immunization of Mice with a Recombinant Adenovirus Vaccine Inhibits the Early Growth of *Mycobacterium tuberculosis* After Infection

**DOI:** 10.1371/journal.pone.0008235

**Published:** 2009-12-09

**Authors:** Edward O. Ronan, Lian Ni Lee, Peter C. L. Beverley, Elma Z. Tchilian

**Affiliations:** Nuffield Department of Medicine, University of Oxford, Oxford, United Kingdom; New York University, United States of America

## Abstract

**Background:**

In pulmonary *Mycobacterium tuberculosis (Mtb)* infection, immune responses are delayed compared to other respiratory infections, so that antigen-specific cells are not detected in the lungs earlier than day 14. Even after parenteral immunization with Bacille Calmette Guerin (BCG) or a subunit vaccine, the immune response after *Mtb* challenge is only slightly accelerated and the kinetics of pulmonary *Mtb* growth do not differ between naïve and immunized animals up to day 14.

**Methods and Findings:**

Mice were immunized intranasally with a recombinant adenovirus expressing mycobacterial antigen 85A (Ad85A), challenged by aerosol with *Mtb* and the kinetics of *Mtb* growth in the lungs measured. Intranasal immunization with Ad85A inhibits *Mtb* growth in the early phase of infection, up to day 8. Protection is sustained for at least 7 months and correlates with the presence of antigen-specific activated effector CD8 T cells in the lungs. Antigen 85A-specific T cells respond to antigen presenting cells from the lungs of mice immunized with Ad85A 23 weeks previously, demonstrating the persistence of antigen in the lungs.

**Conclusions/Significance:**

Intranasal immunization with Ad85A can inhibit early growth of *Mtb* because it establishes a lung antigen depot and maintains an activated lung-resident lymphocyte population. We propose that an optimal immunization strategy for tuberculosis should aim to induce both lung and systemic immunity, targeting the early and late phases of *Mtb* growth.

## Introduction

Tuberculosis causes 1.7 million deaths per year worldwide and the emergence of HIV-associated mycobacterial infections, as well as an increasing frequency of multi-drug resistant and extensively drug resistant *M. tuberculosis* (*Mtb*) isolates, reinforces the need to develop new control strategies. Immunization with Bacille Calmette Guerin (BCG) confers a variable degree of protection against disseminated disease in the very young but poor protection against pulmonary disease in older age groups. However, because of its partial efficacy, an attractive strategy is to develop vaccines that can be used as boosters following BCG primary immunization. Because CD4 T cell immunity and IFNγ have been shown to be important for immune control of *Mtb* many of the new vaccine candidates aim to induce a Th1 type CD4 response. While some have been shown to induce protective immunity equivalent to BCG when given alone [1]–[4], these candidates seldom increase convincingly protection over BCG, when they are used as booster vaccines [5]–[8].

In contrast, recombinant adenoviruses expressing *Mtb* mycolyl transferase antigen 85A (Ad85A) have shown good protective effects in several species when given intranasally (i.n.) and also reproducibly increase protection over BCG alone when given as a booster by this route [9]–[11]. Ad85A induces strong CD8 immune responses [10], [12], [13] and in mice, protection induced by i.n. immunization has been shown to be associated with the presence of a large population of antigen-specific CD8 T cells in the lungs [14]. This is in line with more recent evidence that CD8 cells make an important contribution to immune protection against *Mtb* [15]–[17].

A unique feature of pulmonary *Mtb* infection is that the innate and adaptive immune responses occur much later than in other respiratory infections. In mice, no T cell activation occurs in the mediastinal nodes before 9–10 days post-aerosol *Mtb* infection and antigen specific cells are not detected in the lungs earlier than day 14 [15], [18], [19]. Even in mice immunized with BCG, *Mtb* itself or a subunit vaccine, cellular responses are only slightly accelerated. This delay in cellular immune responses is in accord with the kinetics of mycobacterial growth. Thus, over the first 15 days post-infection mycobacterial growth does not differ between naïve and immune mice [20]–[23]. However, the presence of large numbers of CD8 T cells in the lungs of Ad85A i.n. immunized mice suggested that this regime might induce protection by a different mechanism. Here we show that this is the case and mice immunized i.n. with Ad85A suppress mycobacterial growth in the lungs during the first week after *Mtb* aerosol challenge, in contrast to the delayed inhibitory effect of parenteral immunization. As protection against early growth of *Mtb* in the lungs has not been described previously, we further investigated the nature of the cells present in the lungs at the time of challenge and the duration of this form of protection following immunization.

## Results

### Ad85A Immunization Targets the Early Phase of *Mtb* Infection

Parenteral immunization with BCG generates mainly systemic CD4-mediated protective immunity and few antigen-specific cells are found in the airway lumen of the lungs [18], [19], [24], [25]. However, because primary immunization or boosting with Ad85A i.n. induces a large lung-resident CD8 population [10], [12]–[14], we investigated whether this method of immunization affects growth of *Mtb* early after challenge.

**Figure 1:**
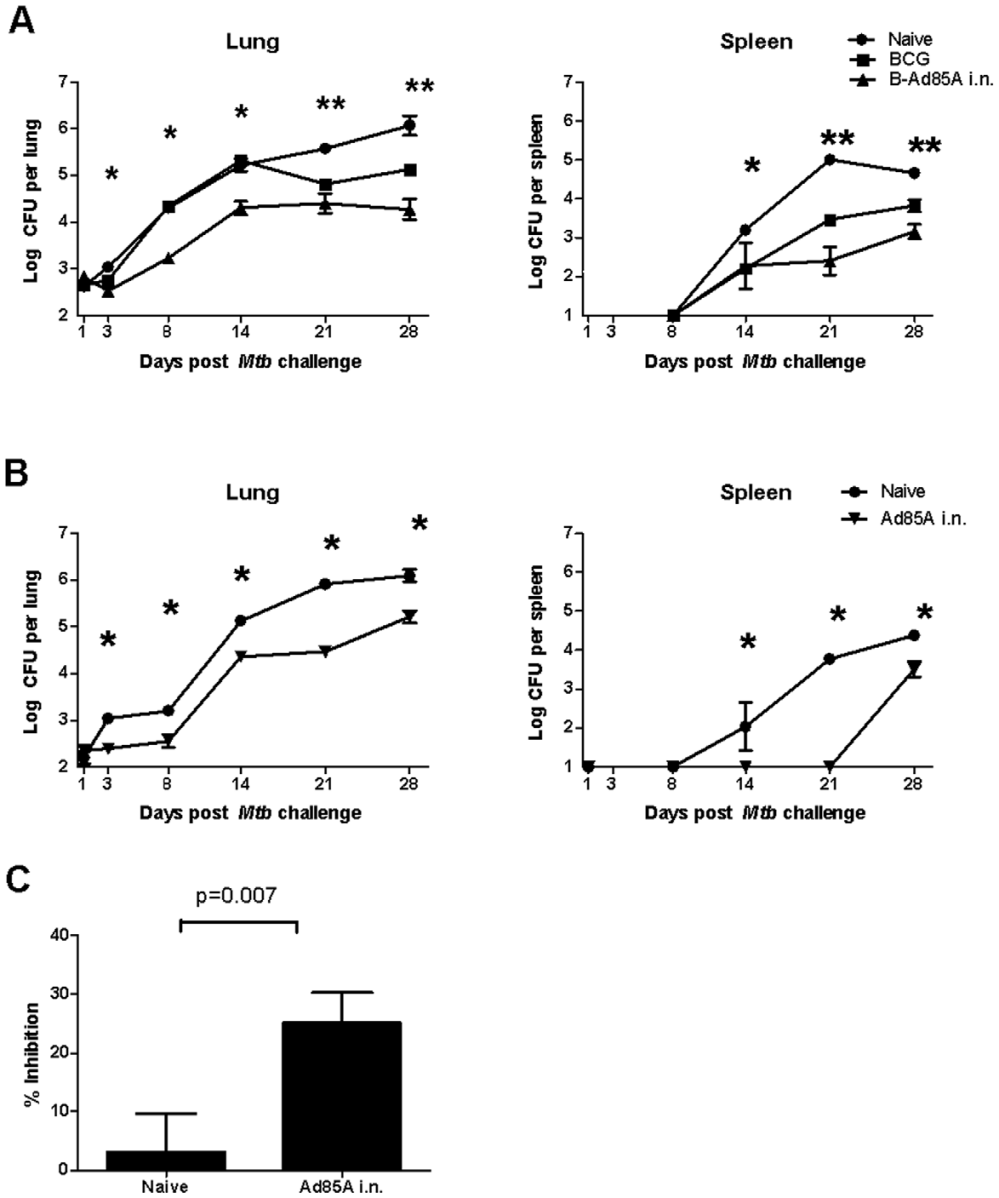
Early control of mycobacterial growth by intranasal administration of Ad85A. BALB/c mice were immunized with BCG or primed with BCG and 10 weeks later boosted with Ad85A i.n. (A) or immunized only with Ad85A i.n. (B) Naïve mice were used as controls. Mice were challenged with *Mtb* by aerosol 4 weeks after the last immunization and sacrificed at days 1, 3, 8, 14, 21 and 28. Lung and spleen CFU were enumerated. Results are expressed as the mean counts of 5–7 mice per group. * p<0.05 Ad85A or B-Ad85A immunized mice versus Naïve or BCG, **p<0.05 for B-Ad85A versus Naïve, BCG versus B-Ad85A, BCG versus Naïve. Similar results were obtained in a repeat experiment. Standard deviations are small, so that the error bars are within the symbols when not visible. (C) Inhibition of *Mtb* growth in macrophages by lung cells *in vitro*. Murine peritoneal macrophages were infected with *Mtb* and co-cultured with lung cells from naïve mice or mice immunized with Ad85A i.n. 3 weeks previously. Inhibition of mycobacterial growth was determined after 3 days. Results are expressed as the mean % inhibition +/− SEM of four experiments with four mice per group.

Mice were immunized with BCG alone or primed with BCG and boosted with Ad85A i.n. 10 weeks later. Four weeks after the last immunization they were challenged with *Mtb* by the aerosol route and mycobacterial load was quantitated at early time points. Strikingly, in mice boosted with Ad85A the mycobacterial load in the lungs does not increase up to 8 days after challenge, while naïve and BCG-immunized animals show a steady increase in *Mtb* CFU from day 3 onward and no difference in lung mycobacterial load until day 21 post-challenge (**Fig. 1A**). Thus mice boosted with Ad85A i.n. control *Mtb* by a different mechanism from BCG-only mice. However, in order to exclude the effect of priming by BCG, we also tested *Mtb* growth in animals immunized with Ad85A i.n. only. We observed similar early inhibition of *Mtb* CFU, confirming that immunization with Ad85A i.n. inhibits early *Mtb* growth, in contrast to BCG, the effect of which only becomes apparent 21 days after *Mtb* infection (**Fig. 1B**).

Because of the early suppression of *Mtb* growth in the Ad85A immunized mice, we examined the ability of lung cells from mice immunized with Ad85A i.n. to inhibit *Mtb* growth in macrophages *in vitro* (**Fig. 1C**). Lung cells from animals immunized i.n. with Ad85A inhibit growth of *Mtb* on average by 25.2+/−5.2% compared to lung cells from naive mice (3.1+/−6.5%, p = 0.007).

### Protection After Intranasal Administration of Ad85A Is Long Lasting

Protective efficacy of tuberculosis booster vaccines is most often tested at 4 to 6 weeks after boosting, at or near the peak of the immune response. However, for a vaccine to be useful it is important that durable protective memory is established. We therefore examined protection against *Mtb* aerosol challenge at 4 and 24 weeks post-i.n. immunization with Ad85A.

**Figure 2:**
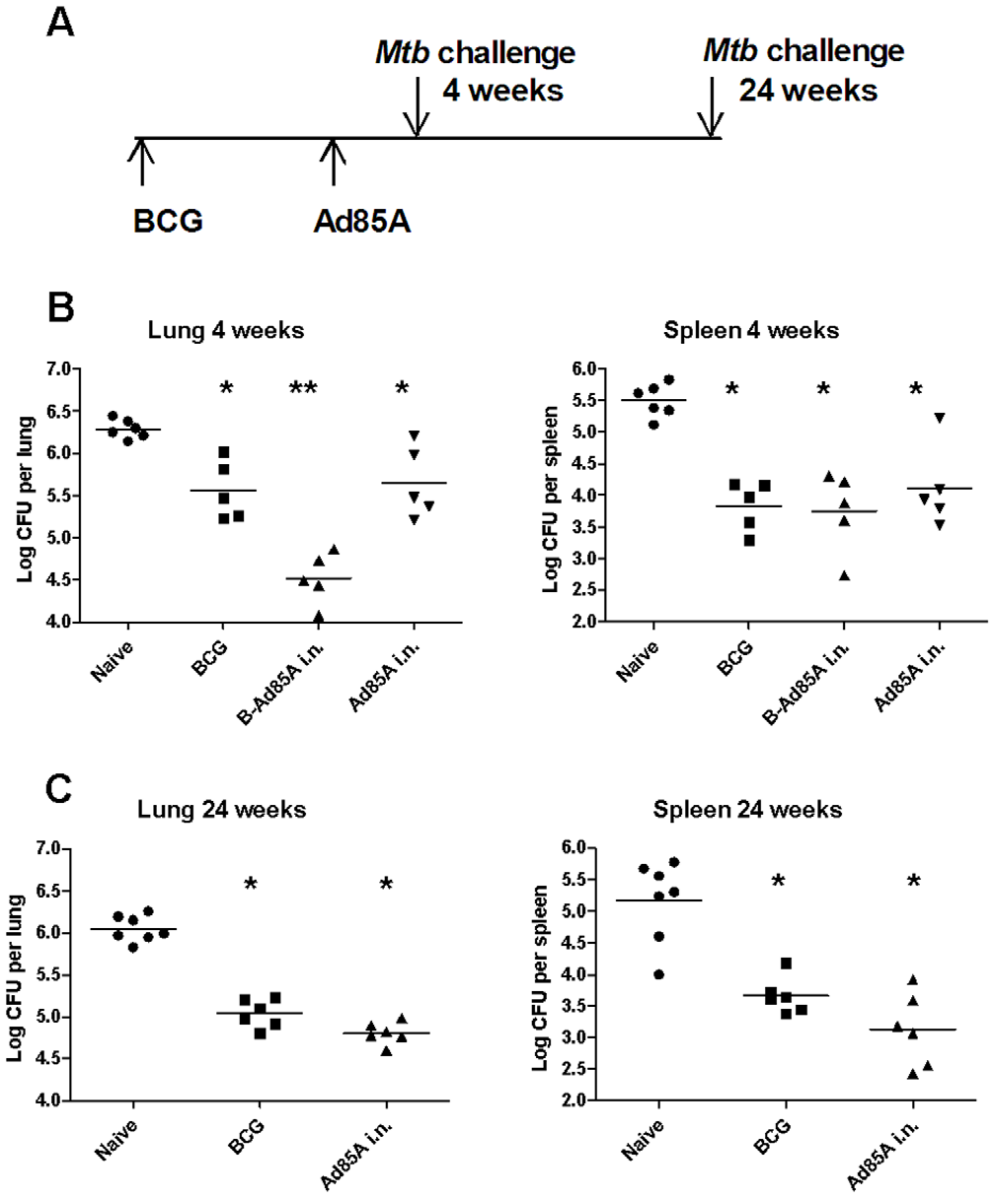
Intranasal administration of Ad85A increases protection at 4 and 24 weeks. (A) Time line of the experiment. BALB/c mice were immunized with BCG, or primed with BCG and 10 weeks later boosted intranasally with Ad85A (B-Ad85A i.n.), or immunized only with Ad85A i.n. Naïve mice were used as challenge controls. Mice were challenged with *Mtb* by aerosol 4 (B) or 24 (C) weeks after the boost. Deposition in the lungs was measured 24 h after challenge and was ∼200 CFU/lung. Mice were sacrificed 6 weeks later and lung and spleen CFU enumerated. Symbols show CFU counts of individual mice and the horizontal line indicates the mean. * p<0.05 versus Naïve, **p<0.05 versus BCG. Similar results were obtained in two other experiments where the lungs and spleens were harvested at 4 weeks post *Mtb* challenge.

When mice are challenged by aerosol 4 weeks after immunization with Ad85A i.n., *Mtb* lung CFU are reduced by ∼1 log compared to naïve animals, a similar reduction to that in mice immunized with BCG. Ad85A i.n. on its own also reduces the *Mtb* load in the spleen to a similar extent as BCG only (∼2 logs) (**Fig. 2B**). As previously described, the protective effects of BCG priming followed by an Ad85A i.n. boost are approximately additive in the lungs, although in this experiment no additive effect was seen in the spleen [10], [14].

BCG-induced protection is long-lasting in mice, so it was of interest to examine whether this was the case for Ad85A i.n.. **Figure 2C** shows that the protective effect in lung and spleen afforded by Ad85A on its own is sustained 24 weeks after i.n. immunization (∼1 log in lung and ∼2 logs in spleen, p = 0.03). A second experiment with *Mtb* challenge at 30 weeks post-immunization showed sustained reduction in CFU in BCG-immunized (lung ∼1 log reduction compared to naive p = 0.004, spleen ∼1 log p = 0.02) or Ad85A i.n.-immunized mice (lung ∼0.6 log p = 0.004, spleen ∼0.6 log p = 0.004). No reduction of CFU was observed when Ad85A was administered i.d. either on its own or as a booster following BCG (**Table 1** and [14]).

**Table 1:** Comparison of protection after different methods of pulmonary infection with *Mtb*.

**Lung**		**I.N.**		**I.T.**		**aerosol**
**Naive**	6.45	±0.33	6.58	±0.41	6.36	±0.22
**B**	5.53	±0.39*	5.36	±0.60	5.75	±0.32*
**BA i.d.**	5.41	±0.36*	5.07	±0.23*	5.35	±0.63*
**BA i.n.**	4.62	±0.14**	3.20	±0.57**	4.95	±0.48**
**Spleen**		**I.N.**		**I.T.**		**aerosol**
**Naive**	4.71	±0.25	4.40	±0.38*	5.62	±0.19
**B**	4.25	±0.34*	4.45	±0.25*	3.95	±0.16*
**BA i.d.**	4.15	±0.43*	4.10	±0.43*	3.22	±0.83*
**BA i.n.**	3.21	±0.36**	2.45	±0.72**	3.38	±0.62*

BALB/c mice were immunized with BCG, or primed with BCG and 8 weeks later boosted with Ad85A given intradermally (BA i.d.) or intranasally (BA i.n.). Naïve mice were used as controls. Mice were challenged with *Mtb* 4 weeks after the last immunization intranasally (I.N.), intratracheally (I.T.) or by aerosol. Deposition in the lungs was measured 24 h after challenge and was ∼1000 CFU per lung after I.N., 620 CFU after I.T. and 880 CFU after aerosol *Mtb* challenge. Mice were sacrificed 4 weeks later and lungs and spleen CFU enumerated. Data represent the mean +/− SD from 6–8 mice per group.

*p<0.05 versus naïve.

**p<0.05 versus BCG and BA i.d.

Although we did not test the protective effect of BCG priming and Ad85A i.n. boosting at 24 or 30 weeks, we did so at 14 weeks and the protection afforded is still significantly better than BCG alone (∼0.5 log reduction in lung compared to BCG, p = 0.016; spleen ∼1.4 log, p = 0.009, data not shown) However, further experiments to establish the durability of the additional protection provided by intranasal boosting with Ad85A need to be performed.

Several different methods have been used for pulmonary challenge with *Mtb* in mice and the challenge dose may also affect the outcome of experiments [10], [12], [13]. We therefore tested whether protection afforded by i.n. immunization with Ad85A is seen after both low (∼200 CFU, **Fig. 2**) or high dose (880 CFU) *Mtb* aerosol challenge (**Table 1**) or when the mice are challenged with *Mtb* by the intratracheal or i.n. routes (**Table 1**). Irrespective of the method, or dose of *Mtb* used for pulmonary *Mtb* challenge, i.n. boosting with Ad85A significantly reduces lung and spleen CFU compared to BCG alone, confirming earlier studies [6], [9], [10], [12], [13].

These results confirm previous findings that i.n. administration of Ad85A induces robust protection when given on its own and provides additional protection when given as a booster after BCG. We also show that protection is sustained for at least 30 weeks.

### Lung Antigen 85A Immune Responses

**Figure 3:**
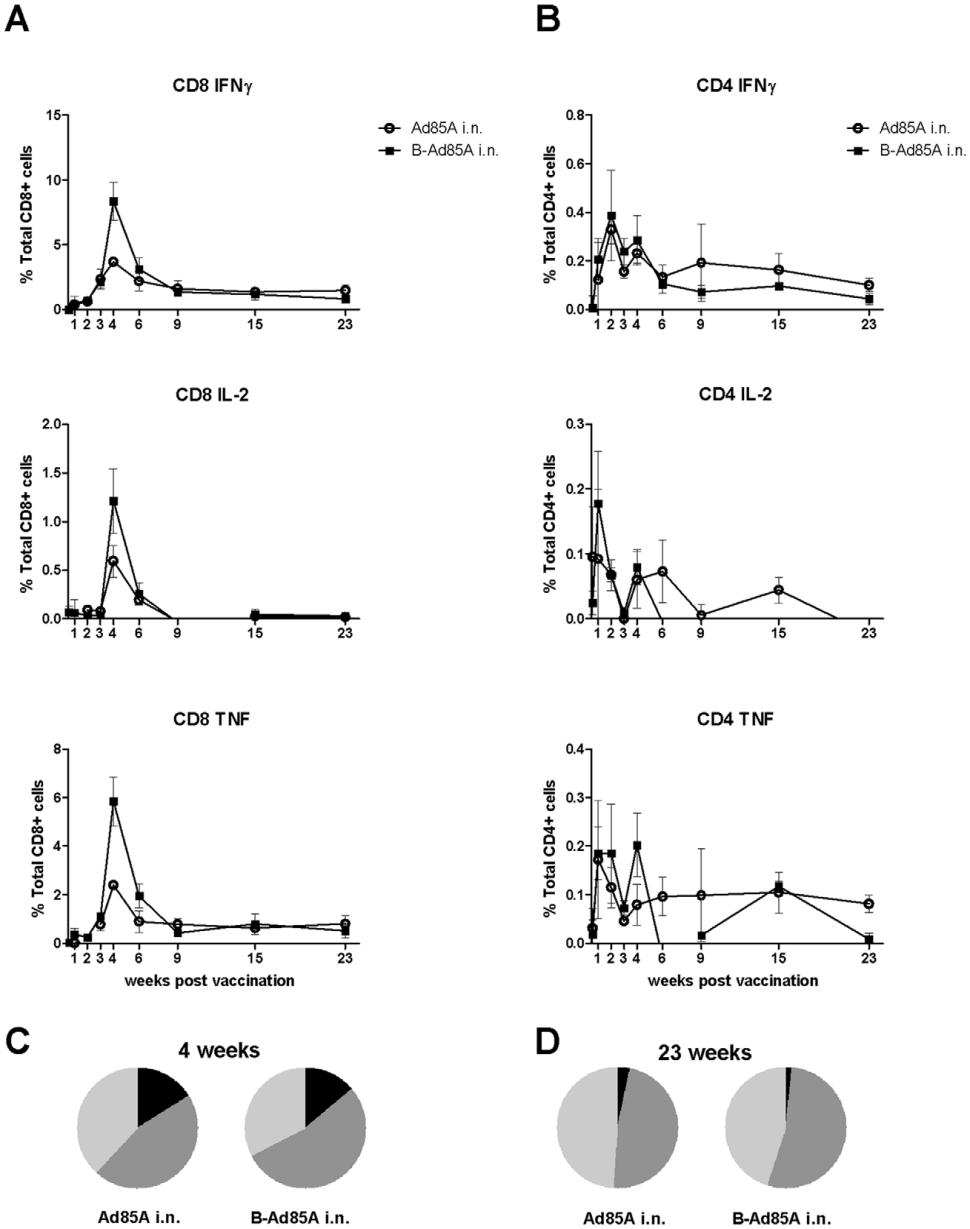
Cytokine responses of lung T cells to antigen 85A. Mice were immunized with Ad85A i.n. (Ad85A i.n.) or primed with BCG and boosted 8 weeks later with Ad85A intranasally (B-Ad85A i.n.). Lung cells were isolated at the indicated times after the last immunization and stimulated with pooled 85A peptides for 6 hours. The frequencies of IFNγ, IL-2 and TNFα producing cells were determined by flow cytometry on CD8 (A) and CD4 (B) gated cells. Pie charts indicate the proportions of single (light grey), dual (dark grey) and triple (black) CD8 producers of IFNγ, TNFα and IL-2 in the lungs of mice 4 (C) and 23 (D) weeks after immunization. Results are expressed as the mean +/− SEM of three/four mice per group, representative of two independent experiments.

Because protection after i.n. administration of Ad85A correlates with the presence of antigen-specific cells in the lung at 4 weeks [14], we investigated whether these cells are present in the lung later after immunization. We performed intracellular staining for IFNγ, IL-2 and TNFα on lung cells stimulated with a pool of peptides covering the whole protein sequence of antigen 85A (**Fig. 3A**). The peak of the response is 4 weeks after immunization. At this time point mice primed with BCG and boosted with Ad85A i.n. show a greater response than mice immunized with Ad85A i.n. only, implying that immunization with BCG primes for a subsequent booster response to antigen 85A. Previously we observed no priming effect of BCG on the 85A response but here we used BCG SSI as opposed to Pasteur and a different interval between priming and boosting, 8 weeks as opposed to 10 weeks in our previous study [14]. In a separate repeat experiment with a 10 week interval between the prime and boost, no priming effect of BCG on the 85A response was observed, suggesting that the priming effect is weak and inconsistent.

In BCG primed, Ad85A i.n. boosted animals at the peak of the response, ∼8% of the lung CD8 T cells produce IFNγ in response to the pool of 85A peptides. As previously shown many fewer CD8 cells produce IL-2, while TNFα production is similar to IFNγ [14]. The CD8 response declines from 8% to ∼1% of lung CD8 cells at 23 weeks. CD4 responses are relatively low and 0.4% and 0.1% of the CD4 cells are antigen-specific at 4 and 23 weeks respectively (**Fig. 3B**). A low response (0.5% of CD8 cells) is detected in the spleen and peripheral blood following i.n. vaccination.

Because triple cytokine-producing cells have been suggested to be important for protection against some chronic infections, including *Mtb* [26], we analysed the proportions of single (1+), double (2+) and triple (3+) cytokine-producing CD8 85A- specific cells at 4 and 23 weeks (**Fig. 3C and D**). There are some 3+ cells at 4 weeks, as there is some IL-2 production. However these disappear rapidly and at week 23 there are mainly 2+ and 1+ cells. No difference in the frequency of cells producing different numbers of cytokines is detected between the prime and prime-boost regimes. Nor was there any change over time of the median fluorescence intensity of staining for intracellular IFNγ, IL-2 and TNFα in animals immunized with Ad85A i.n. only.

Parenteral i.d. immunization induces a long-lived splenic immune response which lasts for at least 23 weeks. The magnitude and duration of the antigen-specific CD8 response in the spleen of i.d. Ad85A immunized animals is very similar to the lung response in Ad85A i.n. immunized animals, with 3% of splenic antigen-specific cells at the peak of the response and 1% at 23 weeks, for both the prime and prime-boost regimes. Despite this, Ad85A i.d. immunization does not reduce mycobacterial load on its own, nor does it further reduce the load when administered as a boost, confirming that the induction of a strong systemic immune response does not correlate with protection in this mouse model [14].

**Figure 4:**
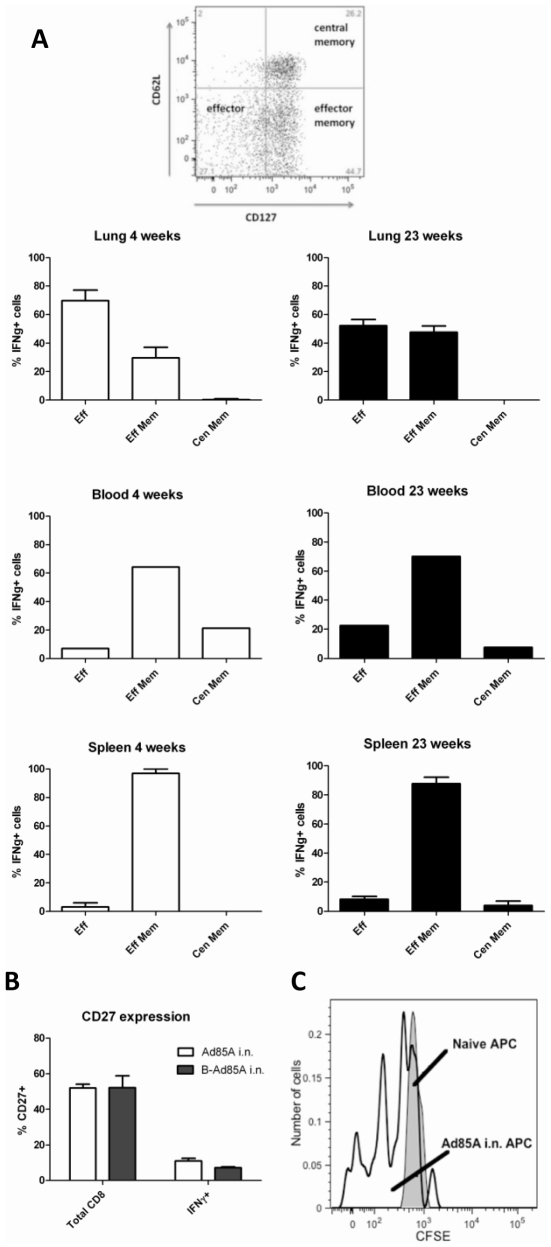
Phenotype of antigen-specific cells following intranasal immunization with Ad85A. Mice were immunized as described in the legend to Fig. 2 and lung cells stained for CD8, IFNγ, CD62L and CD127 (A) or CD27 (B). All results are from Ad85A i.n. mice at 4 and 23 weeks. Similar data were obtained from B-Ad85A i.n. mice. Results are expressed as the mean +/− SEM from three or four mice except for blood where the PBMCs from three/four mice were pooled. Similar results were obtained from two independent experiments. (C) Persistence of antigen 85A in the lungs. Cells from the lungs of mice immunized 23 weeks previously with Ad85A i.n. (open histogram) or naïve control mice (shaded histogram) were used as APCs and co-cultured with CFSE-labelled 85A-specific splenic T cells. Cells were recovered at day 5 and stained with H-2L^d^ 85A peptide _70–78aa_ (MPVGGQSSF) tetramer. The histogram shows the CFSE profile of 85A tetramer positive CD8 cells. The result of one representative experiment of three is shown.

Having shown that i.n. immunization with Ad85A establishes a stable, long-lived protective immune response against infectious challenge, we further characterised the antigen-specific T cells. Antigen-specific IFNγ^+^CD8 cells were identified as effector (CD62L^−^CD127^−^), effector memory (CD62L^−^CD127^+^) and central memory cells (CD62L^+^CD127^+^) [27] (**Fig. 4A**). In the lung, antigen-specific cells are predominantly effectors, 70% and 52% respectively at 4 and 23 weeks post-immunisation and the remaining cells are effector memory phenotype (30% and 48% respectively). In contrast, although there were very few antigen-specific cells in extra-pulmonary sites of these i.n. immunised animals, those in the spleen and blood are predominantly effector memory phenotype at 4 and 23 weeks (**Fig. 4A**).

When mice are immunized i.d. with Ad85A, the splenic and blood antigen-specific cells have the same phenotype as the much smaller number of cells present in these organs after i.n. immunization (**Fig. 4A**). Therefore irrespective of the route of immunization, blood and splenic antigen-specific cells are less activated than those in the lung, confirming previous observations that cells that home to non-lymphoid tissues display an activated phenotype [28], [29].

Because down-regulation of CD27 has been associated with activation and persistent antigenic stimulation, we analysed CD27 expression in lung cells [30]. **Fig. 4B** shows that antigen-specific cells in the lung lack expression of CD27 compared to total CD8 cells, suggesting that the lung might contain antigen 85A, even at 23 weeks post-immunisation.

In summary, persistent antigen-specific responses are detected in the lungs following i.n. administration of Ad85A. Long-term memory in the lungs of i.n. immunised animals is biased towards effector type CD8 T cells producing mainly IFNγ and TNFα.

### Presence of Antigen 85A in the Lungs

As we detect antigen-specific cells in the lungs at 23 weeks post-immunisation and because they lack CD27 surface expression, we next examined whether antigen is still present in the lungs. We used lung cells isolated 23 weeks post-Ad85A i.n. immunisation as APCs to stimulate T cells isolated from the spleens of animals immunized i.d. with Ad85A (**Fig. 4C**). The responding T cells were labelled with CFSE and co-cultured with unlabelled lung APCs. As more than 90% of the CD8 antigen-specific cells recognise the dominant H-2L^d^ antigen 85A peptide _70–78aa_ (MPVGGQSSF), this tetramer was used to detect antigen-specific cells responding to the lung APCs. After 5 days CFSE intensity of tetramer positive cells was analysed and compared to the same cells co-cultured with lung APCs from naïve animals. After co-culture with APCs from Ad85A i.n. animals, tetramer binding cells undergo several cell divisions, whereas cells co-cultured with lungs cells from naïve animals do not, indicating that 85A antigen, capable of inducing T cell proliferation, is present in the lungs 23 weeks post-immunization (**Fig. 4C**). Although we did not include an empty adenovirus control, since only the tetramer positive splenic T cells proliferated and not other activated CD8 cells in the splenic T cell population, it appears that proliferation is antigen specific, rather than a response to inflammatory cytokines.

## Discussion

Pulmonary *Mtb* infection is unlike other respiratory infections because innate and adaptive immune responses to *Mtb* are delayed. T cell activation in the mediastinal nodes only occurs 9–10 days post-infection, while antigen-specific cells are not detected in the lungs until day 14 [18], [19]. Although not fully understood, this delay may be due to low antigen dose (50–200 mycobacteria), naturally low inflammatory responses in the lung or active inhibition of antigen presentation by *Mtb* [18]. Whatever the reasons, a consequence is that mechanisms for promoting entry of systemic antigen-specific cells, such as up-regulation of VCAM-1, are not activated until two weeks post-infection [31]. It is therefore understandable that parenteral immunization against *Mtb* provides no protection against initial growth of mycobacteria following pulmonary challenge [20]–[23]. Here we demonstrate for the first time that a protective Ad85A vaccine administered i.n. exerts its effect by suppression of mycobacterial growth during the first week after *Mtb* infection, in contrast to the delayed inhibitory effect of parenteral vaccines.

Ad85A and recombinant adenoviruses containing other antigens of *Mtb* administered i.n. are highly protective in mice and induce large populations of lung-resident CD8 T cells [4], [9], [12], [14]. These remain largely confined to the lungs because of endothoracic recirculation [29]. Even six months after immunization with Ad85A i.n, there is a significant population of antigen-specific cells in the lungs and their presence correlates with protection against *Mtb*. More than 50% of lung 85A-specific CD8 cells are of effector phenotype, even at 23 weeks post-immunization and the majority have down-regulated CD27, a phenotype that is thought to indicate continual exposure to antigen and correlates with expression of granzyme B and perforin [30]. The proliferation of 85A-specific T cells in response to lung cells of mice immunized six months previously with Ad85A, provides direct evidence for persistence of antigen, confirming other data showing persistence of adenoviral sequences up to 2 years post-intramuscular immunization [32].

Although cells from the lungs of mice immunized with Ad85A i.n. reduced *Mtb* CFU of infected macrophages *in vitro* we do not know the exact mechanism by which early inhibition of growth *in vivo* is achieved. Indeed, there is little clarity regarding the cellular and molecular mechanisms of the immune responses that limit *Mtb* growth after infection of naïve animals, or mediate vaccine-induced protection. BCG induces mainly CD4 T cells producing TNFα and IFNγ as well as multifunctional cells [24], [26] and an extensive literature indicates the importance of CD4 T cells in immunity to *Mtb* [18]. We do not know whether lung cells from BCG-immunized mice inhibit *Mtb* growth in macrophages but it will be of interest to compare them with lung cells from Ad85A-immunized mice in future experiments. However, it is clear that CD8 T cells can also play a role in protection [15], [17], [18]. Administration of Ad85A i.n. induces a strong and durable antigen 85A-specific lung CD8 response of effector and effector memory phenotype and these CD8 cells may function by producing IFNγ and by targeting infected cells for cytolysis, using several cytolytic pathways [17]. It is also possible that immunization with Ad85A i.n. recruits or activates other leucocytes such as neutrophils and macrophages that could contribute to inhibition of early growth of *Mtb*. Evidence that IL-17 is associated with protective immunity in cattle and mice may suggest that this is the case [6], [19], [22]. Further work will elucidate the exact mechanisms and genes involved in early control of *Mtb* growth in the lungs.

Recombinant adenoviruses administered i.n. are a highly effective means of inducing mucosal immunity, CD8 T cells and protection against *Mtb*, but there is some controversy as to the efficacy of parenteral immunization with similar recombinant vectors. We have previously shown that i.d. boosting with Ad85A does not significantly increase protection over that afforded by BCG alone in mice challenged by aerosol [14], but because others have used different methods we compared aerosol, intratracheal and intranasal pulmonary challenge. In all cases, an i.d. Ad85A boost after BCG priming did not significantly increase protection, although there was a trend toward lower CFU in the boosted mice. On the other hand, intramuscular (i.m.) boosting with Ad85A in mice and guinea pigs clearly provides at least transient protection [11]. In mice, it has been shown that Ad85A i.m. immunization elicits transient lung immune responses [13] but the responding cells are not localised in the airway lumen, in contrast to the situation following i.n. immunization [12]. The transient nature of pulmonary protection in these experiments is therefore most likely because cells that enter the lungs after parenteral immunization do not remain there in the absence of antigen. This explanation is supported by experiments in which recombinant 85A protein was introduced into the trachea of mice immunized parenterally with recombinant Ad85A, leading to more sustained protection against pulmonary challenge [12].

Several recent studies have demonstrated that it is possible to generate protective immunity equivalent to that of BCG by parenteral immunization. The most successful regimes employ recombinant proteins given repeatedly in adjuvants [3], [7]. However, there is little evidence that such regimes provide consistently increased protection over BCG when used as boosters. We suggest that the principal reason why immunization with Ad85A i.n. consistently provides additive protection when given as a booster after BCG is that it generates a depot of antigen in the lungs that both maintains an antigen-specific population and ensures that the antigen–specific cells remain in an activated state, able to mediate early protective immune responses to pulmonary *Mtb* challenge. Nevertheless it remains possible that some parenteral immunization methods may also induce cell populations that might provide protection in the lungs early after pulmonary challenge. A recent study in rhesus macaques provides some encouragement, as repeated immunization with different recombinant adenoviruses expressing SIV Gag resulted in potent, durable and functional CD8 responses at multiple mucosal surfaces [33], [34].

In conclusion, our data confirm and extend previous results showing that i.n. administration of Ad85A provides robust protection against pulmonary tuberculosis in BALB/c mice. We show that protection is sustained for at least 7 months and correlates with the presence of an antigen-specific CD8 T cell response in the lungs. Intranasal immunization with Ad85A prevents an increase in *Mtb* CFU in the lungs during the first week after pulmonary challenge. In contrast, following parenteral immunization with BCG or an ESAT6 peptide subunit vaccine, inhibition of mycobacterial growth is only seen later after *Mtb* infection (>15 days) [22], [23]. Therefore we propose that an optimal immunisation strategy should target both phases of mycobacterial infection. Intranasal immunization targets the early phase because it establishes a lung antigen depot and an activated lung-resident lymphocyte population. Parenteral immunization with a protective antigen (BCG or ESAT6) establishes systemic immunity, but systemic *Mtb*-specific cells only enter the lungs when the challenge *Mtb* has caused sufficient inflammation to recruit circulating leucocytes [20]–[23].

Current prime-boost strategies assume that BCG primes for *Mtb* antigen(s) present in the booster vaccine and therefore generate a more powerful protective response, but even the parenteral prime-boost regimes currently entering clinical trials do not provide statistically significantly better protection than BCG alone [5]–[7]. In contrast we suggest that parenteral Ad85A i.n. and BCG have an additive effect because they target early and late phases of *Mtb* growth, not because BCG primes for an 85A response. Formal proof of this will come from experiments using *Mtb* subunit vaccines containing different antigens to generate separate lung and systemic immune responses. Meanwhile we propose that mucosal and parenteral immunization, targeting the early and late phases of infection, most likely via different antigens, is a rational strategy to improve protective immunity to *Mtb*.

## Materials and Methods

### Ethics Statement

All animal experiments were approved by the animal use ethical committee of Oxford University and fully complied with UK Home Office guidelines.

### Construction of a Recombinant Adenovirus Expressing Antigen 85A

The human tissue plasminogen activator leader sequence was fused to the coding region of *Mtb* 85A (nt 99797 to nt 98910, lacking the leader sequence) (Geneart), subcloned into pDONR221 vector, then recombined into E1/E3 deleted AdHu5 (pAd/CMV/V5-Dest) (Invitrogen) following the manufacturer's instructions. A replication-deficient AdHu5-85A recombinant virus clone was amplified in *E.coli*, digested with PacI and transfected into 293A cells to produce an adenoviral stock. Bulk virus was produced by infecting 293A cells with the stock, then purified (PureSyn), and quantitated by spectrophotometry. This construct demonstrated similar immunogenicity and protection against aerosol *Mtb* challenge as another recombinant Adenovirus-85A construct [14].

### Animals and Immunizations

All experiments were performed on 6–8 week old female BALB/c mice (Harlan Orlac). BCG (SSI) was administered subcutaneously in the left hind footpad (2×10^5^ colony forming units (CFU)/30 µl). For i.d. boosting, mice were anaesthetized and immunized with 25 µl in each ear (2×10^9^ virus particles (vp) of Ad85A per mouse), and for i.n. boosting allowed to slowly inhale 50 µl of 2×10^9^ vp of Ad85A. Mice were also immunized with Ad85A without prior BCG priming.

### Pulmonary *M. tuberculosis* Challenge

Four to 30 weeks after Ad85A immunization, aerosol or intratracheal challenge with *Mtb* (Erdman strain, CBER/FDA) was performed as described [14], [35]. For i.n. infection, anaesthetized mice were allowed to inhale 50 µl of *Mtb*. Deposition in the lungs was measured 24 h later. Mice were sacrificed at the indicated times; the *Mtb* load in spleens and lungs was determined by plating 10-fold serial dilutions of tissue homogenates on Middlebrook 7H11 agar plates (E & O Laboratories Ltd).

### Cell Isolation and Flow Cytometry

Lungs were perfused with PBS, minced and digested with 0.7 mg/ml collagenase type 1 (Sigma) and 30 µg/ml DNase 1 (Sigma) for 45 mins at 37°C, then passed through a cell strainer, washed and monuclear cells selected by density centrifugation on Lympholyte (Cederlane). Spleens were passed through a cell strainer and erythrocytes were lysed with RBC lysis solution (Qiagen). Peripheral blood was collected in heparinized tubes from 3–4 mice per group, diluted with DMEM and purified over Lymphoprep (Axis-Shield). Isolated lymphocytes were cultured in DMEM+10% FCS, L-glutamine, 2-mercaptoethanol, penicillin and streptomycin and stimulated with either a pool of 66 15-mer peptides overlapping by 10 amino acids encompassing the entire 85A protein, or with the dominant CD4 (Ag85A_99–118aa_ TFLTSELPGWLQANRHVKPT) and CD8 (Ag85A_70–78aa_ MPVGGQSSF and Ag85A_145–152aa_ YAGAMSGL) peptide epitopes [36], [37] at 2 µg/ml (Peptide Protein Research) for 6 hours at 37°C, with the addition of GolgiPlug (BD Biosciences) 1 hour post-stimulation.

For intracellular cytokine staining, the Cytofix/Cytoperm kit (BD Biosciences) protocol was employed. Cells were washed, treated with Fc block (eBiosciences), then stained with combinations of CD4 (clone RM4–5), CD27 (LG.7F9), CD127 (A7R34), CD62L (MEL-14), IFNγ (XMG1.2), IL-2 (JES6-5H4), TNFα (MP6-XT22) (eBioscience) or CD8 (53-6.7) depending on the experiment. The H-2L^d^ 85A peptide _70–78aa_ (MPVGGQSSF) tetramer (NIH Tetramer Facility) for the dominant 85A epitope in H-2^d^ mice was used in some experiments to enumerate antigen-specific CD8 cells. Cells were fixed with PBS+1% paraformaldehyde, run on an LSRII (BD Biosciences) and analysed on Flow Jo (Tree Star Inc). 3–4 individual mice were analysed per group. Cytokine producing cell frequencies and numbers presented are after background subtraction of an identically gated population of cells from the same sample, incubated without peptide.

### Detection of Antigen 85A in Lung Cells

Lung cells were prepared from mice immunized with Ad85A i.n. 23 weeks previously. CD8 T cells were removed using CD8 Microbeads and the remaining antigen presenting cells (APCs) were plated in 48-well plates (8×10^5^ cells per well in 1 ml of RPMI+10% FCS, L-glutamine). To generate CFSE-labeled responder cells, CD4 and CD8 T cells were positively isolated (Miltenyi) from single cell suspensions of splenocytes of mice immunized with Ad85A i.d. 20 weeks earlier, then labeled with CFSE (Invitrogen), washed and added to the APCs at a concentration of 2×10^5^ cells per well. APCs and responder T cells were incubated for 5 days at 37°C. Recovered cells were labeled with H-2L^d^ 85A peptide _70–78aa_ (MPVGGQSSF) tetramer, CD19 (1D3), CD8 and 7AAD to exclude dead cells. Proliferation of the tetramer specific population of CFSE-labeled CD8 T cells was measured on an LSRII.

### Mycobacterial Growth Inhibition Assay

The assay was performed as described [14]. Peritoneal macrophages were plated at 15,000 cells per well in U-bottomed 96 well microtiter plates and incubated at 37°C overnight. Non-adherent cells were removed and adherent cells were infected with *Mtb* overnight at a MOI of 5∶1. Lung cells isolated from Ad85A i.n.-immunized or naïve animals were added at an effector to target ratio of 12∶1. After 72 hours co-culture, 0.2% saponin was added for 1 hour to release *Mtb*. Viable organisms were quantitated by plating on 7H11 agar. Results are expressed as % inhibition of mycobacterial growth calculated by the formula, C-E/C x100, where C is the number of CFU in cultures of infected macrophages in medium alone and E is the number of CFU in co-cultures containing lung cells from either naïve or Ad85A i.n.-immunized animals.

### Statistical Analysis

All results are representative of at least two independent experiments with similar results. Data were analysed using the non-parametric Mann-Whitney test.
